# Fifteenth Annual Meeting of the Miss. Valley Association of Dental Surgeons

**Published:** 1859-03

**Authors:** 


### FIFTEENTH ANNUAL MEETING OF THE MISS. VALLEY ASSOCIATION OF DENTAL SURGEONS.

### DISCUSSIONS.

1. *Treatment of Inflamed or sensitive dentine.*

Dr. TAYLOR said he would not make a speech, but would allude to the use of a single agent—chloride of zinc. He preferred to use it in crystals, or paste, rather than in solution. This, he believed, was common to the profession. The point he wished to get at was the length of time the chloride should remain in the tooth. He formerly applied it from day to day, but was then often disappointed in its use, finding the tooth often as sensitive as ever. Now he applies it, in substance, and lets it remain ten or fifteen minutes, and seldom has difficulty in excavating afterward.

Dr. H. R. SMITH remarked that when we wait a day after applying the chloride, we usually find the tooth more sensitive than before the application. He agreed with Dr. Taylor as to the proper way to use it, and thought that for sensitive dentine it is, perhaps, our best local remedy.

Dr. TAYLOR had recently used a paste made of creosote and chloride of zinc combined. His method was to leave it in the tooth ten or fifteen minutes—usually till the pain produced by it has subsided. The use of it thus far had been highly satisfactory. He was of opinion that the chloride loses strength by keeping. He inquired if it destroyed the sensibility of normal dentine as readily as it did that of diseased. He thought it did not, and gave a case to prove the correctness of his opinion.

Dr. BONSALL thought the chloride does not change its properties by keeping, but is merely diluted by its absorption of water.

Dr. ULREY said that his experience with the chloride had not corresponded with that of Drs. Taylor and Smith. When he applied it one day, he did not find the sensibility increased the next.

Dr. TAFT regarded chloride of zinc as one of our best therapeutic agents. It was very good in some cases, and of no use in others. In many cases sensibility would be increased by its use. He had applied it five or six times in an hour, and still the dentine remained sensitive. But in most instances in which it was indicated, a single application would be followed by satisfactory results. He preferred to have it in a crystaline form; and, with proper care, he said, it could be easily kept in this state. He had a bottle of it, which had stood on his case for several months, which was still crystalized. His method of using it was to apply it in substance, and let it remain till the pain subsides. If but a thin lamina be sensitive, a single application will answer the purpose. He believed that it not only acts as an escharotic, but also paralyzes the nervous fibrils beyond the range of its escharotic action. This paralysis gradually subsides, and, of course, then we find a return of the sensibility. He referred to a case in the treatment of which he repeatedly applied the chloride to a large cavity in a molar tooth. Every part of the cavity was highly sensitive, and no relief was obtained by the treatment. He filled the cavity with sponge gold, making firm pressure. The sensibility remained for some time afterward, but was now gone.

Dr. TAYLOR remarked that he had no doubt that the sensibility lost or destroyed by the chloride was, in due time, usually restored; and this fact, he thought, showed a recuperative power in the dentine which we had not previously been willing to concede. In the case just referred to, he maintained that Dr. Taft had paralyzed the nervous fibrils by the pressure used in filling the tooth. This doctrine he had once discarded, but had come back to it.

Dr. BONSALL remarked that though he used escharotics and astringents, yet he preferred the pressure of a hard cut, where it could be endured.

Dr. WEBSTER remarked that he seldom uses chloride of zinc. He preferred the use of creosote protected, and retained in the cavity, by a pledget saturated with sandarac. He usually let the creosote remain in the cavity two or three days.

Dr. H. R. SMITH after using the chloride of zinc, was in the habit of applying a mixture of sulphuric ether and chloroform, equal parts by bulk,—this he applied to the gum as well as to the cavity in the tooth. He had used with success, as an application to dentine, the preparation of aconite mentioned by Dr. Teft, at the late meeting of the American Convention.

Dr. WATT spoke of the general action of escharotics. They act by virtue of their affinity for one or more of the constituents of the living tissue to which they are applied. The chloride of zinc has a strong affinity for water, especially in its crystaline form. A part of its escharotic action was due to this property. But it has also a strong affinity for albuminous tissues; and this it would manifest in solution, as well as when solid. With this view it was plain that its escharotic action would be much reduced by dissolving it. The whole action of this, and of all escharotics, is *chemico-vital.* The destruction of vitality in one part induced increased vitality in the subjacent parts. He thought it was on this principle that a *return* of, or even increased sensibility was to be explained. He referred to the first article in volume 10 of the *Dental Register*, for a further elucidation of his views on this subject.

Dr. RICHARDSON remarked that he seldom uses any thing to allay the sensibility of dentine; but, when he does, he usually prefers the chloride of zinc. The most sensitive points were usually just beneath the enamel. When he found the patient flinching, he usually sharpened his instrument, and cut deeper and quicker. Under this treatment the tooth became less sensitive, though he was at a loss to know just why. He thought, perhaps, the patient bore it better on finding that the operator was determined. He generally ascertained by a few experimental cuts, in what direction the least pain was produced, and then cut as much as practicable in that direction. He had used aconitine with doubtful results.

Dr. Taft stated that hard pressure, with a burnisher over the entire walls of the cavity, would allay the sensibility. The application would be attended with some pain, but so would any other efficient mode. When a small point between the enamel and dentine was sensitive, he said that to rap with a blunt instrument on the enamel, immediately over the point, would relieve it for a time; but, as it would soon return, a repetition would often be necessary. This, of course, he said, would not be applicable to all situations.

Dr. Compton had not used the chloride of zinc with satisfaction. He had usually succeeded best on the plan mentioned by Dr. Richardson.

Dr. Taft stated that, in some cases, the constitution would not admit of any successful *local* treatment. In many such cases the tooth may be filled, but it remains sensitive, the patient still suffers, and the tooth finally dies. Sometimes all the teeth were thus sensitive. If so, the constitution must be treated before filling. Local treatment also, will be of advantage, but must not be relied on to the exclusion of the other.

Dr. Bonsall said he regarded constitutional treatment as of great importance in such cases.

2. *Mechanical Dentistry.*

4. *To what extent are atmospheric plates applicable to partial sets of teeth?*

(By consent, these two subjects were considered together. —Rep).

Dr. Taylor inquired whether deep or flat arches were the more troublesome, in adapting suction plates to them.

Dr. Ulrey remarked that in regard to deep arches, he had nothing new to offer. In swaging plates to fit them, he used a number of dies. He would prefer an arch, neither very deep nor flat; but would rather have a flat than a very deep one. It was less trouble to fit the flat; but, when the flat was perfect, it would adhere more firmly than the deep one. In reply to a question of Dr. Taylor, he said that when the plate rests on the ridge, but does not touch the palate, he makes new dies and stretches the plate by hammering it down.

Dr. TAYLOR said that when the arch is very deep he preferred to make partial dies, and swage down the palatal portion first. In such cases he would cut out the cavity, that he might be able to see that the adaptation was perfect, and then swage a piece of plate to cover it, and solder it on.

Dr. BONSALL spoke of a case in which the absorption was very great,—the jaws were so far apart, that ordinary work would be too heavy. The arch was very deep; and the ridge seemed to be entirely cartilaginous. It had been found necessary to use springs. The patient's gold plates are now both lined with a gutta percha base, to atone for the absorption. He had suggested the use of the vulcanite base, but had promised the patient to report to her again.

Dr. WEBSTER inquired if, when the suction was good, the weight would be perceived.

Dr. BONSALL replied that a light piece would often be partly sustained by the muscles and other soft parts of the mouth, independent of the action of atmospheric pressure.

Dr. WEBSTER thought an ounce would fall as quick as a pound.

Dr. TAYLOR reported a case in which the arch was very shallow, and the lips attached almost to the center of the ridge. The lower jaw was very prominent, the natural teeth striking outside of those of the upper jaw. The patient, however, was not willing that the artificial teeth should antagonize in the same manner. Several dentists had made work for her, himself among the number; but none had succeeded in giving a practical job. He finally held out the upper lip, and took an impression with it in that position. He curved the border of the plate, extending it so high up that the curve rested firmly against the junction of the lip and gum. He used very long teeth for the upper jaw, and set them outside of the alveolar border, and mounted them by the continuous gum method. The plate adhered firmly, the features were restored, and the whole affair was satisfactory. The patient had since gone to Chicago; and he had just heard that Dr. Allport had put her up a cheoplastic set.

Dr. VANEMON inquired how far the lower ridge protruded beyond the upper.

Dr. TAYLOR replied, one inch.

Dr. VANEMON said he had a similar case which he managed in the same way.

Dr. RICHARDSON—Do we generally find a very large under jaw accompanied by a very small upper one?

Dr. TAYLOR—Yes.

Dr. BONSALL reported the case of a patient seventy-five years old, having the six natural front teeth in the lower jaw. The lower maxilla was very prominent, and the teeth protruded. The upper jaw was small, and very flat. After failing by the ordinary mode, he made a *very* light plate, and put but eight teeth on it; and the patient now eats very well with it. He thought that work of the ordinary weight would have failed, without more lower teeth to antagonize with.

Dr. RICHARDSON had such a poor opinion of partial lower sets that he very seldom put them in. In adapting upper sets, if one or both bicuspids, on each side below, were still standing, the work could be made quite satisfactory; and he advised patients not to have lower teeth inserted.

Dr. ULREY inquired what is the advice of the profession, if the six lower front teeth remain and are healthy, and the patient applies for an upper set.

Dr. TAYLOR said he would pull them out,—or, if not, he would insert lower teeth.

Dr. BONSALL would not extract them, if the patient was young.

Dr. RICHARDSON said that if the posterior portions of the jaw curved so as to incline the plate to press forward, rather than backward, he would insert a lower plate. To Dr. Taylor—he said he would prefer to have the wisdom teeth in, if healthy and standing favorably, as they would prevent the plate from sliding back.

Dr. ULREY would save all sound healthy teeth—even two in the upper jaw. He reported a case in which there were two good molars on each side of the upper jaw, which the patient wished to have extracted, to make room for a full upper set. He refused to extract them. She finally determined that, if he would not, she would get some one else to remove them, and then have him (Dr. U.) to insert the artificial set. He removed them, but was now sorry that he did so. He asked for the views of the members in regard to the matter.

A decided majority declared their opposition to extraction, under the circumstances.—REP.

Dr. TAFT remarked that deep arches gave most trouble, especially if the rugæ were soft. The difficulty was to get a perfect conformation of the plate. The size and situation of the rugæ, and their extent, modified the case. In all such difficult cases he preferred to take the impression in plaster. The plaster used for impressions should be selected with care. We should obtain that which does not suddenly pass from the plastic to the solid state. Then, properly stirring it till it is nearly ready to set, it should be applied, while it is still quite soft. In these difficult cases, he would prepare, and use if necessary, a number of dies, and, if he made a cavity plate, he would cut out the portion covering the cavity, swage down and solder on the covering.

Dr. VANEMON remarked that when a plate fits well, the patient would experience no senstion of weight. The pressure upward would be equal to the downward pressure, otherwise the plate would not be sustained. A light piece, he maintained, would drop as quickly as a heavy one. He supposed that all would concede that weight was no objection to under sets. In answer to the President, he stated that he makes none but continuous gum work.

Dr. TAFT thought that weight was worthy of consideration. A weight might be held by atmospheric pressure, to the under side of one scale of a correct balance ; and it would certainly indicate its presence, by the tilting of the beam.

Dr. VANEMON did not think this was a correct illustration of the principle under consideration.

Dr. RICHARDSON thought weight had much to do with the adhesion of plates ; as very light plates would often adhere, when the fit was very imperfect,

Dr. H. A. SMITH wished to know if there was a perfectly reliable mode of obtaining a natural "bite," as a guide for antagonizing the teeth.

Dr. WEBSTER thought the under jaw could not be forced too far back. We had, therefore, a reliable guide in one direction.

Dr. VANEMON said he had seen cases in which the lower jaw could be forced farther back than its natural and comfortable position. His own course was to let the patients sit erect, in an easy position, and have them shut the mouth, while the attention was devoted to something else. He then retained the jaw in the position it assumed, and had the mouth closed to the proper degree.

Dr. COMPTON remarked that he uses continuous gum work almost exclusively. His platinum plates are not very heavy, as he believes as good work can be made on comparatively light plate. He solders with pure gold, in a muffle. He makes partial sets on gold, with a small platinum plate stamped over it. He sets the teeth, lifts off the platinum plate, puts on the gum, and solders in a muffle. He then attaches the platinum to the gold plate by rivets.

Dr. ULREY inquired if there was not danger of galvanic action.

Dr. COMPTON said there was none. He would add farther, that every thing must be clean about it; and pure gold must be used for solder.

Dr. WATT said that no galvanic action could ensue from the contact or presence of two metals in the mouth, unless the fluids of the mouth were capable of acting chemically on at least one of them. As neither the gold nor the platinum would corrode in the mouth, no galvanic current would be establish

Dr. ULREY reported a case in which the patient's lips were blistered, by wearing a gold plate above, and a silver one below.

Dr. WEBSTER reported a similar case and said that when the silver plate was brightened, the action was stronger, till it again became corroded.

Dr. S. B. SMITH had put in but three silver plates in the last ten years; he would not put two metals in the mouth, as in the cases referred to by Drs. U. and W. The effect might not be very perceptable at first; yet bad results were likely to follow. He had, in some instances, put in gold, at the price of silver work rather than do it.

Dr. TAFT wished to know in what proportion of cases the members present use the atmospheric pressure plates for partial sets.

Dr. ULREY said he had but recently resorted to suction plates for partial sets—used them now in about five per cent of his cases.

Dr. VANEMON supposed he used them in about 15 per cent of his cases.

Dr. WEBSTER—In not more than one case in a hundred.

Dr. TAYLOR—In pieces of more than four teeth, about 50 per cent; with less than four teeth, about 15 or 20 per cent.

Dr. H. A. SMITH—About 25 per cent.

Dr. S. B. SMITH—Uses them in a very small proportion of cases. As the injury from clasps was chemical, he maintained that it might be mostly prevented, by proper attention to cleanliness.

Dr. Wardle has used them in about 25 per cent of his cases—would use them oftener if the patients would pay prices that would justify his doing so. He further remarked that he usually took impressioms for partial sets in plaster. Formerly he had been troubled with the plaster drawing from the impression cup; but he had so constructed a cup, as to effectually prevent such an accident.

Dr. Richardson said that the difference in cost, between clasp, and suction plates, was more apparent than real. The clasp plates were narrower; but they required greater thickness. In a plate for three, or four teeth, there could not be more than two or three pennyweights. Consequently, the difference in cost was a matter of small moment. He was daily becoming more in favor of atmospheric plates, and supposed that, in the last six months, he had inserted fifty per cent. of his partial sets on this plan.

Dr. Watt seldom used clasps. Inserted fully ninety per cent. of partial sets on suction plates. Would rather insert them than clasp plates, at the same price.

Dr. Taft said the per cent. stated by Dr. Watt was none too high as regarded their practice when together. He scarcely ever uses clasps. He regarded this as a vastly important question. Thousands of teeth were destroyed annually by the use of clasps. As to difference in price, that was not worth taking into the account. He would rather make the suction work, at the same price. He could make three suction jobs, while he could make two clasp ones. He preferred plaster impressions, and regarded Dr. Wardle's impression cup as an important improvement, for taking partial impressions in plaster. This method was just as applicable to "ragged" jobs as to full arches. In such cases he would make the main plate thin, and would thicken the narrow necks passing between the teeth. He considered that clasps were never safe; as people could not be induced to take sufficient care to prevent injury to the teeth to which the clasps were attached.

Mr. J. T. Toland and assistants now brought in the flasks, containing the pieces of "coralite base" which were packed yesterday, these being now vulcanized. While they were removing them from the flasks and plaster, the remarks of the members took a very wide and interesting range, to which we can not pretend to do justice.

Dr. Taft exhibited a full set of teeth, mounted on the "coralite" base, which had been worn a few weeks with entire satisfaction, and which, as yet, showed no symptoms of change from the action of the fluids of the mouth.

Dr. Watt, on behalf of Thomas Coke Wright, Esq., of Xenia, exhibited to the association a forcep and two gum-lancets, which were made by Mr. Wright's father, and used by him as early as 1787, and as recently as December, 1858, the venerable operator being over ninety years old, when he extracted the last of his own teeth, with his own instruments—his favorite "*'peasers.*" As he was now ready to resign his instruments, he donated them to the museum of the Ohio Dental College. Mr. W., throughout, discarded the turn-key.

Dr. Taylor gave an interesting sketch of some of the early scenes of his professional life, in one of which he made the acquaintance of Mr. Wright above referred to, and operated for some members of his family, he having previously become acquainted with his son, T. C. Wright, Esq. [We believe Dr. T. promised us a sketch of these early scenes for publication.—Rep.]

Dr. Bonsall said that, before we thought of dies for swaging, he had seen a plate made by pressing several thin layers of gold successively on a plaster model, and then soldering them together.

Dr. Lindsey remarked that he makes his suction plates without cavities. [Dr. L. did not arrive till after the principal discussions on Mechanical Dentistry were over.]

3. *Treatment of dental periostitis, and alveolar abscess.*—Dr. Taft remarked that the ordinary indications of periostitis are familiar to all. The principal are soreness, and elongation of the teeth. The latter symptom is modified by the character or shape of the root. In treating it, it is necessary to consider the character and state of the constitution, the predisposing, and exciting causes, and also whether the case be acute or chronic. All these considerations would modify the treatment. In ordinary cases, local treatment would suffice. The local treatment would consist, mainly, of depletion and counter-irritation. If the case were of long standing, more heroic treatment would be necessary than for a recent case. Constitutional treatment would often be required. The Dentist might conduct this himself, or refer the case to a physician. To induce counter-irritation, he frequently made an incision in the gum, opposite the end of the root, and cauterized it with creosote.

Dr. Richardson said that one result of periostitis had given him a great deal of trouble. He referred to the swelling of the gums and face. This was often found in connection with the front teeth, if they were filled when the pulps were exposed, or nearly so. Pain and swelling were not always relieved, in such cases, by removing the filling. Suppuration has not yet taken place; and it is desirable to have the case terminate in resolution. He would like to hear from the members in regard to the proper treatment of those cases. He did not like warm applications—thought they tended to promote suppuration. He had used cold—even ice—with advantage. He thought stimulating applications increased the difficulty.

Dr. Taft remarked, in explanation, that, in his remarks, he had reference only to cases in which the inflammation was confined to the periosteum.

Dr. Richardson replied that that far, they mainly agreed.

Dr. Ulrey thought that there was danger of producing necrosis of the alveolar process, by the free application of creosote to the gum.

Dr. Taft stated that, in regard to cold and warm applications, he sometimes used the one, and sometimes the other, with benefit. He could not give a clear explanation of the reasons why he preferred the one to the other in any given case. The various pathological conditions were to be taken into consideration.

Dr. Lindsey said that he usually gives an emetic, and then steams the face with the vapor of vinegar.

Dr. Watt remarked that, when there was an increased quantity of blood in the part, yet no obstruction in the vessels, the application of cold, by contracting the vessels, would dispel the surplus quantity of blood, and thus relieve the pressure; but, if the vessels were obstructed, their contraction would only increase the pressure, and consequently the pain, while the application of warmth, by dilating the vessels, might allow the blood to flow on without obstruction, and thus afford relief. If this view were correct, he maintained that the feelings of the patient would furnish a reliable guide in selecting the class of applications.

Dr. Taylor said that Dr. Watt had advanced his ideas. He was in the habit of using cold, or warm applications, as the feelings of the patient might direct. He said it was important to notice the first symptoms of periostitis. For instance, if the pulp is dead, and the canal filled, a little uneasiness might be felt, scarcely amounting to actual pain. That was the proper time to treat the case. He generally cautioned his patients in regard to such cases, and directed them, in case of difficulty, to use cold applications, if not painful, and to take a brisk cathartic. He referred to a case in his own mouth, several years ago. The pulp of a lateral incisor died, and periostitis ensued. He took an emetic, and was relieved. The disease returned about once a year, and was relieved, a second and a third time, by the same remedy. In a fourth attack, however, it failed. In the latter case, the inflammation had terminated in suppuration, in the former, it had not. The pulp, he said, was generally dead before periostitis is established; and relief might often be obtained by taking out the plug, and opening the pulp cavity. As to poulticing, he said physicians usually adopt it; yet he thought the swelling was increased by it. Instead of poultices, he was in the habit of applying roast figs to the gums.

Dr. Bonsall said he was opposed to the use of poultices. He thought they tended to point the abscess externally, in case of suppuration. He recommended roast figs, as mentioned by Dr. Taylor. When the abscess showed a tendency to point externally, he applied pressure on the outside, by a cap of sheet lead, supported by a binder. He thought much could be done in this way.

Dr. Richards said that when it became evident that an abscess would point externally, he used the lancet to open it; as the scar would be less unsightly, than if it were left to open spontaneously.

Dr. Lindsey said he would always extract the tooth, if the abscess showed symptoms of opening externally.

Dr. Ulrey said that, when it must open externally, he would use the lancet, and also extract the tooth; as the discharge would be likely to continue, while the tooth remained.

Dr. H. R. Smith said we could usually tell when there was danger of periostitis. In its earliest stages, he would apply to the gums a mixture of ether, chloroform and ammonia. He would also use constitutional treatment—an emetic in a bad case. He would, if necessary, use derivations freely, and, with a straight bistoury, would cut down from the neck of the tooth till the point of the instrument was opposite the end of the fang, and would keep this incision open. Then, if suppuration took place, it would point to this.

5. *Anæsthetic agents, use of, in the extraction of Teeth.*—Dr. Taft said he abandoned ether and chloroform two years ago, on account of the danger incident to their use. If he used either of them now, he would take ether alone. More deaths from chloroform were reported now than formerly. Local anæsthesia, by congelation, was not objectionable on account of danger, but it was, to say the least, very inconvenient. It was also painful, if not very gradually applied. He had used it but little; and, in his practice, it had often produced severe pain, but had sometimes almost entirely relieved the pain of extraction. If the process and gum were very thick, he considered it impracticable; if very thin, so that congelation to the end of the fang could be induced, it might be of use. He had experimented some with the galvanic current. He didn't pretend to know how it operates. He was at a loss to know how to account for the difference of opinion among the profession. Some say they always succeed,—others always fail. [Dr. T. told a humorous anecdote, in regard to a failure with the battery; but, as all who know us are aware, we are opposed to anecdotes—don't tell them, and won't let Dr. T., at least in this report.—Rep.] He said, further, that the strength of the current must be tested with each patient, before applying it to the tooth. The shocks should be felt to the elbows; and then the force should be slightly diminished. Sometimes, however, it might be used much stronger than this. In general, it should not be unpleasantly strong. The finer the vibrations of the machine, the better; and either pole might be connected with the forcep. Intense pain, he said, was sometimes felt when the tooth was affected with periostitis. He said that we may be so awkward, in the use of the battery, as to cause pain unnecessarily, but as long as in nine cases out of ten, the patient stated that there was little or no pain, he was bound to believe that the electric current had some influence. He referred to a patient who had waited for three years to have some fourteen or fifteen teeth extracted. Hearing of this method, she came to have *one* removed by way of experiment. She had all removed, and said there was no pain. A properly constructed battery, he said, was easily arranged—could be charged and ready for use in half a minute.

Dr. Bonsall said he had not used the battery; but he reported a case of a patient of his who had very firm teeth. Knowing that Dr. Taft was experimenting with the battery, he advised the patient to have the experiment tried on his teeth. The battery was applied, and, after taking out several teeth without any complaint from the patient, one seemed to hurt badly. The patient insisted that something had got wrong in the arrangement of the apparatus. They maintained that all was right, and concluded that a failure had occurred; but, on further examination, they found that the forceps was disconnected. It was readjusted, and all went off satisfactorily. He said, further, that he does not use chloroform. He had seen several cases where the health was bad after its use, as he believed, from the nervous shock produced by it. He was, however, very favorably impressed with electricity, since seeing it used.

Dr. H. A. Smith spoke of a case experimented on in the College Infirmary. The patient had several teeth to be extracted. The battery was used on each alternate tooth, the patient not knowing that the connection was broken. The difference in favor of the battery was very obvious. In this case, he used the gum lancet, in connection with the battery, and, he believed, with benefit. He was opposed to the use of chloroform in dental operations.

Dr. Keely had not used the battery. He did not administer ether or chloroform himself, but had frequently operated when chloric ether was administered by a physician.

Dr. Watt said that, if the operation to be performed was very severe, and there was nothing in the constitution of the patient to contra-indicate it, he would use a mixture of ether and chloroform. The severity of the operation lessened the danger. When he could get at the teeth, with the patient in a recumbent posture, he preferred that position for the administration of anæsthetics. He thought there was less danger. If the symptoms became alarming, he always suspended the administration of the anæsthetic. As to the battery, he had experimented a little with it, and was highly pleased with the results. He supposed it lessened the pain by rousing the nerves concerned in the operation up to the

"fighting point." In a battle, or street fight, a man may receive a severe—even a fatal wound, and yet feel no pain when it is inflicted.

Dr. BONSALL said his own experience confirmed the last remark of Dr. W. In resisting a mob, he once received a hurt which kept him in bed some time; yet he did not feel it when inflicted, but fought on, till the riot was quelled.

Dr. COLLINS said that if the horizontal position is safer, in using chloroform, it was probable that the danger in its use arises from loss of tonicity in the vessels, and not from a disorganization of the blood.

Dr. LINDSEY said he was an early user of ether, but did not like it. Now he uses chloroform, and likes it. He gives it to all that want it, and to some that don't. He referred to a case in which great prostration occurred; but the patient was sustained by artificial respiration. He would never administer chloroform to a lady, without the presence of a third person.

Nos. 6, 7, and 8, were not discussed, for want of time. The reporter would state, in conclusion, that the discussions assumed, at this meeting, more of a conversational character than usual. Practical ideas seemed to be in demand. Much of the most interesting and profitable portions of the proceedings can not be reported for want of the specimens and appliances by which they were illustrated. No report can do justice to an old-fashioned meeting of the MISSISSIPPI VALLEY ASSOCIATION. W.

